# Early Pulmonary Complications following Total Knee Arthroplasty under General Anesthesia: A Prospective Cohort Study Using CT Scan

**DOI:** 10.1155/2016/4062043

**Published:** 2016-03-16

**Authors:** Kai Song, Zhen Rong, Xianfeng Yang, Yao Yao, Yeshuai Shen, Dongquan Shi, Zhihong Xu, Dongyang Chen, Minghao Zheng, Qing Jiang

**Affiliations:** ^1^Department of Sports Medicine and Adult Reconstruction, Drum Tower Hospital, Medical School of Nanjing University, Zhongshan Road 321, Nanjing, Jiangsu 210008, China; ^2^Joint Research Centre for Bone and Joint Disease, Model Animal Research Center (MARC), Nanjing University, Xuefu Road, Nanjing, Jiangsu 210032, China; ^3^Centre for Orthopaedic Research, School of Surgery, University of Western Australia, M508, 35 Stirling Highway, Crawley, WA 6009, Australia

## Abstract

*Purpose.* Postoperative pulmonary complications (PPCs) are common after major surgeries. However, the number of studies regarding PPCs following total knee arthroplasty (TKA) is limited. The aim of this study was to determine the incidence of early PPCs following TKA by computed tomography (CT) scan and to identify associated risk factors.* Methods.* Patients, who were diagnosed with osteoarthritis or rheumatoid arthritis and underwent primary TKA at our institution, were included in this prospective cohort study. Patients received a standard procedure of TKA under general anesthesia. Chest CT scan was performed during 5–7 days postoperatively. Univariate analysis and multivariate logistic regression analysis were employed to identify the risk factors.* Results.* The total incidence of early PPCs following TKA was 45.9%. Rates of pneumonia, pleural effusion, and atelectasis were 14.4%, 38.7%, and 12.6%, respectively. Lower body mass index and perioperative blood transfusion were independent risk factors for PPCs as a whole and associated with atelectasis. Postoperative acute episode of hypoxemia increased the risk of pneumonia. Blood transfusion alone was related to pleural effusion.* Conclusions.* The incidence of early PPCs following TKA was high. For patients with relevant risk factors, positive measures should be adopted to prevent PPCs.

## 1. Introduction

Postoperative pulmonary complications (PPCs), defined as respiratory failure, pneumonia, pleural effusion, atelectasis, pneumothorax, or aspiration pneumonitis, are the leading cause for ICU admission, prolonged hospital stay, increased cost, and higher mortality after surgery [1]. The incidence of PPCs ranges from 5.8% to 39.6% depending on different surgical procedures [2]. Advanced age, smoking, chronic obstructive pulmonary disease (COPD), congestive heart failure, higher American Society of Anesthesiologists (ASA) classification, prolonged surgery, general anesthesia, and emergency surgery increase the risk of PPCs [3, 4].

Total knee arthroplasty (TKA) is widely employed in the treatment for end-stage osteoarthritis (OA) and rheumatoid arthritis (RA). This surgical procedure allows for the improvement in patients' clinical symptoms and quality of life, but complications related to TKA represent a major problem for orthopedists. Pneumonia is one of the most common complications after total hip and knee arthroplasty [5]. It is the major cause of readmission and death after surgery [6, 7]. In addition, atelectasis and pleural effusion are also common after total joint arthroplasty, which may adversely affect the outcomes of patients [6, 7]. However, despite the prevalence and subsequent severe consequences of pulmonary complications following arthroplasty, the number of studies regarding these events is limited and the majority of them only focused on pneumonia. Moreover, PPCs are usually underestimated, because sometimes they exhibit only slight symptoms or none at all, which may cause the difficulty in diagnosis. Computed tomography (CT) scan can provide more accurate information about the early postoperative complications. The aim of this study was to determine the incidence of early PPCs, including pneumonia, pleural effusion, and atelectasis, following TKA by CT scan and to identify associated risk factors.

## 2. Methods

### 2.1. Patient and Data Collection

Patients, who were diagnosed with OA or RA and underwent lateral primary TKA at our institution between September 2013 and July 2014, were scheduled for this prospective cohort study. Patients were excluded from this study if they had previously known pulmonary disorders. For patients who received staged TKAs for both knees during this period, only the first procedure was included. This study was approved by our institutional review board (IRB) and written informed consent was obtained from each participant.

After admission, we recorded each participant's demographic information including gender, age, body mass index (BMI), coexisting illnesses, and current smoking history. Coexisting illnesses included diabetes, hypertension, malignance, heart diseases, anemia, and stroke. Steroid use within 7 days before surgery was also recorded. All surgeries were performed using medial parapatellar arthrotomy with tourniquet, by or under the supervision of an experienced surgeon. All patients received general anesthesia during operation. Intravenous infusion of 500 mL of lactated Ringer's solution was given prior to anesthesia induction, which is subsequently conducted with etomidate, midazolam, vecuronium, fentanyl, and propofol. Continuous infusion of propofol, remifentanil, and cisatracurium was employed intraoperatively. Ventilation was performed with oxygen/air and controlled to maintain end-tidal CO_2_ at 30–35 mmHg. Cefuroxime was used as antibiotic prophylaxis. Recommended thromboprophylaxis, consisting of chemical and mechanical prophylaxis, was employed for each patient. Rehabilitation program started on the first postoperative day under the guidance of a physical therapist. Chest CT scan was performed during 5–7 days after surgery. CT results were interpreted by two radiologists and reviewed by a senior radiologist. Pulmonary complications detected by CT were recorded, such as pneumonia, atelectasis, and unilateral and bilateral pleural effusion. Pneumonia was diagnosed by the infiltration, consolidation, or cavitation on the CT images, combined with clinical manifestations including fever, increased respiratory secretions, cough, and dyspnoea. Subsequently, blood tests and spectrum culture were conducted to further confirm the diagnosis of pneumonia. Pleural effusion was diagnosed by the finding of fluid accumulated in the pleural space. Atelectasis on CT images showed opacification accompanied by overinflation in the adjacent lung.

Patients' preoperative hemoglobin levels, intraoperative blood loss, and postoperative drainage were recorded. If patients received blood transfusion during perioperative period or developed hypoxemia after surgery, such information was also collected. Hypoxemia was diagnosed when a pulse oximetry reading was lower than or equal to 90%.

### 2.2. Data Analysis

According to the results of CT screening, the incidence of pneumonia, atelectasis, and pleural effusion following TKA was calculated. Variables, such as age, gender, BMI, coexisting illness, steroid use, smoking history, preoperative hemoglobin level, the diagnosis of RA, operation and tourniquet time, intraoperative blood loss, postoperative drainage, perioperative transfusion, and postoperative hypoxemia, were introduced into the univariate analysis to screen for risk factors associated with PPCs. Categorical variables were compared using chi-square test, and continuous variables (age, BMI, preoperative hemoglobin level, intraoperative blood loss, postoperative drainage, and operation and tourniquet time) were analyzed by *t*-test. Subsequently, factors with *p* value less than 0.05 (BMI, smoking, operation time, and blood transfusion) were introduced into the multivariate logistic regression analysis to determine the independent predictors. A difference with *p* value less than 0.05 was considered statistically significant. According to the rule of ten events per variable in logistics regression [8], four predictors were acceptable for 51 events of PPCs. Collinearity diagnostics was conducted before logistic regression analysis. All of the Variance Inflation Factors (VIFs) were less than 10 (mean VIF: 1.05; range of VIFs: 1.03–1.05), which indicated that multicollinearity was not a concern in this case. Hosmer-Lemeshow (H-L) test and the receiver operating characteristics (ROC) curve were employed to assess the calibration and discrimination of the regression model. All of the analysis was conducted using STATA version 12.0 (StataCorp LP, College Station, TX, USA).

## 3. Results

**Figure 1:**
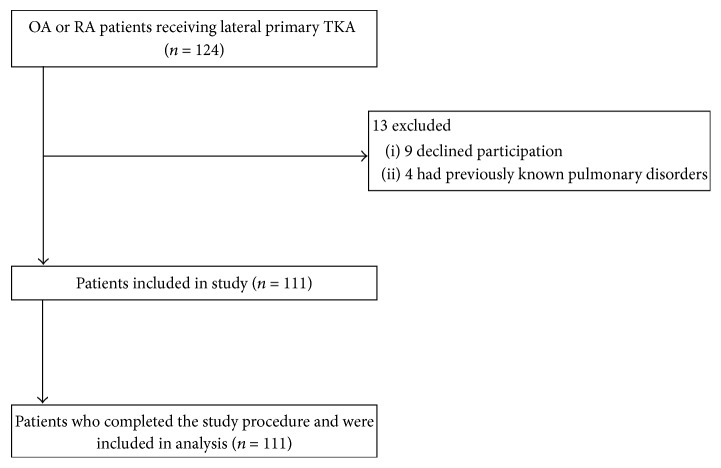
Flowchart of the participants in the prospective cohort study.

From September 2013 to July 2014, 124 patients with OA or RA undergoing lateral primary TKA were accessed for eligibility (Figure 1). Nine patients declined participation. Four patients were excluded because of previously known pulmonary disorders. A total of 111 patients were subjected into the present study, with 91 females and 20 males. All of them completed the study procedure and were included in the analysis.

106 patients received TKA for osteoarthritis and 5 received TKA for rheumatoid arthritis. The mean age was 66.9 years (range: 43–83 years). The mean operation and tourniquet time were 124.2 minutes (range: 90–190 minutes) and 65.0 minutes (range: 47–90 minutes), respectively. Sixty-two patients (55.9%) received autologous and/or allogeneic transfusion during perioperative period. Nine patients (8.1%) experienced acute episode of hypoxemia after surgery, and all of them returned to normal after administration of oxygen. Subsequently, pulmonary embolism was ruled out by computed tomographic pulmonary angiography (CTPA) in these patients.  Table 1 shows the demographic, clinical, and surgical characteristics of the patients.

The total incidence of PPCs documented by CT was 45.9% (*n* = 51). Sixteen patients (14.4%) were diagnosed with pneumonia according to CT images. Among these patients, only one patient developed symptoms of pneumonia, including fever, productive cough, and shortness of breath. He received antibiotics based on susceptibility testing. The others only had slight signs of pneumonia on CT, without relevant symptoms. They only received prolonged cefuroxime rather than targeted antibiotic therapy. Bilateral pleural effusion was the most frequent PPC following TKA (*n* = 36, 32.4%). The incidence of single left and right pleural effusion was 2.7% (*n* = 3) and 3.6% (*n* = 4), respectively. All of the pleural effusions observed were graded as small effusions according to a published method [9]. Atelectasis was found in 14 patients (12.6%), and 13 of them had pleural effusion as well. Therefore, atelectasis might be caused by effusion in most cases.

Taking all kinds of PPCs as a whole, we found that lower BMI (*p* = 0.015), smoking history (*p* = 0.029), longer operation time (*p* = 0.038), and blood transfusion (*p* = 0.004) were related to PPCs in univariate analysis (Table 2). Among these potential risk factors, lower BMI (OR = 0.9, *p* = 0.042) and transfusion (OR = 2.6, *p* = 0.024) were independently associated with PPCs according to multiple logistic regression analysis (Table 3). The area under the ROC curve was 0.738. Calibration test showed that H-L test statistic *χ* ^2^ was 3.06 and that *p* value was 0.930.

Additionally, variables, which were included in the above unifactorial analysis, were also analyzed to screen for risk factors for pneumonia, pleural effusion, and atelectasis. Categorical variables were compared using chi-square test, and continuous variables were analyzed by *t*-test. The value of odds ratio (OR) was given by unifactorial logistic regression. Postoperative acute episode of hypoxemia was associated with pneumonia (*p* = 0.007; OR = 6.0, 95% CI: 1.4–25.5). Perioperative blood transfusion appeared to increase the risk of pleural effusion (*p* = 0.019; OR = 2.6, 95% CI: 1.2–5.8). Lower BMI (*p* = 0.007; OR = 0.8, 95% CI: 0.7–0.9) and blood transfusion (*p* = 0.016; OR = 5.6, 95% CI: 1.2–26.5) proved to be risk factors for atelectasis.

## 4. Discussion

We found that the total incidence of PPCs following TKA was 45.9% according to the results of routine chest CT scan. Rates of pneumonia, pleural effusion, and atelectasis documented by CT were 14.4%, 38.7%, and 12.6%, respectively. Lower BMI and perioperative blood transfusion were independent risk factors for PPCs as a whole and associated with atelectasis. Postoperative acute episode of hypoxemia was associated with pneumonia. Blood transfusion alone was associated with pleural effusion.

Pneumonia increases the risk of readmission and revision surgery for patients undergoing total joint arthroplasty [6, 7, 10, 11]. It is one of the most common causes of death after arthroplasty, conferring a 5.2-fold increase in the risk of mortality [11]. The rate of in-hospital pneumonia following total joint arthroplasty is 0.74–0.86% [5, 11], and this rate was on the rise during the last decade [12]. In the present study, we observed 16 cases of pneumonia documented by CT among 111 patients. The rate (14.4%) is much higher than previous studies. However, only one patient developed symptoms of pneumonia. The others just had slight signs of pneumonia on CT without relevant symptoms. Additionally, general anesthesia, which was used in this study, has been reported to increase the risk of pneumonia after knee arthroplasty [13], by impairing the mucocutaneous defensive barriers through intubation. This may partly explain the relatively high incidence of pneumonia in this study. Hypoxemia has been recognized as a predictor for major pulmonary complications following arthroplasty [14], and we also found its association with pneumonia. Therefore, further evaluation should be taken if patient develops postoperative hypoxemia.

A small amount of pleural effusion is common after major surgeries, and exudates account for a large proportion of these events. Most effusions are asymptomatic and self-limiting, which resolve spontaneously and do not require specific therapy. However, if the pleural effusion expands or remains for weeks, further examination and intervention should be considered. Persistent pleural effusion may increase the risk of atelectasis and pneumonia. The present study showed that 92.9% of atelectasis cases were accompanied with pleural effusion. Atelectasis may be also caused by general anesthesia, during which the production of surfactants on the surface of alveolus is decreased, and subsequent collapse of airway leads to atelectasis. Additionally, the residual effect of anesthetic may cause atelectasis by suppressing deep breath and cough reflex.

The present study was limited by the small sample size, which may lead to the difficulty in determining the risk factors. Smoking history and prolonged duration of operation have been reported as risk factors for PPCs [2, 4, 15], but we found that they did not influence the rate of PPCs independently through multivariate logistic regression analysis. Furthermore, we evaluated the PPCs within only one week after TKA, which may cause the underestimation of the incidence of PPCs. Even though most PPCs develop during the early period after surgery, late lung complications do occur. Additionally, there are several confounders that were not taken into account, such as nutritional status (might be related to low BMI), perioperative fluid therapy (might be associated with pleural effusion), and use of colloids. Since general anesthesia was employed in this study, the incidence of and risk factors for PPCs might be different from studies using regional anesthesia, which might be a potential source of bias.

To the best of our knowledge, this is the first study to screen for the early PPCs following TKA using CT scan. Compared with plain radiographs, CT scan could reveal minor conditions of the lung. However, most of the minor conditions are self-limited and do not require specific treatment. Therefore, CT scan was not recommended as routine examination in patients undergoing TKA, even under general anesthesia.

## 5. Conclusions

Perioperative blood transfusion and low BMI were associated with PPCs in this study, which is consistent with previous study [1]. For patients with these risk factors, measures to prevent PPCs should be adopted, including employing neuraxial anesthesia instead of general anesthesia, early mobilization after surgery, encouraging deep breath, correcting low serum albumin, and restricting fluid transfusion.

## Figures and Tables

**Table 1:** Patient's demographic, clinical, and surgical characteristics (*n* = 111).

Characteristic	Number (%)	Mean (SD)
Gender		
Male	20 (18.0)	
Female	91 (82.0)	
Age, years		66.9 (7.8)
BMI, kg/m^2^		26.5 (3.4)
Diabetes		
Type 1 diabetes	2 (1.8)	
Type 2 diabetes	12 (10.8)	
Hypertension	63 (56.8)	
Malignance	3 (2.7)	
Heart disease		
Coronary heart disease	15 (13.5)	
Arrhythmia	6 (5.4)	
Dilated cardiomyopathy	1 (0.9)	
Rheumatic heart disease	2 (1.8)	
Anemia	5 (4.5)	
Stroke	24 (21.6)	
Steroid use	13 (11.7)	
Smoking	7 (6.3)	
Preoperative hemoglobin level, g/L		130.6 (12.9)
Diagnosis		
OA	106 (95.5)	
RA	5 (4.5)	
Operation time, minutes		124.2 (19.1)
Tourniquet time, minutes		65.0 (11.2)
Intraoperative blood loss, ml		241.0 (96.2)
Postoperative drainage, ml		511.0 (338.1)
Blood transfusion	62 (55.9)	
Hypoxemia	9 (8.1)	

BMI, body mass index; OA, osteoarthritis; RA, rheumatoid arthritis.

**Table 2:** Univariate analysis of the risk factors for PPC.

Variable	Non-PPC (*n* = 60)	PPC (*n* = 51)	*p* value
Male gender (%)	8 (13.3)	12 (23.53)	0.164
Age, years (mean ± standard deviation)	66.5 (8.1)	67.4 (7.5)	0.548
BMI, kg/m^2^(mean ± standard deviation)	27.2 (3.1)	25.6 (3.6)	0.015^*∗*^
Diabetes (%)	9 (15.0)	5 (9.8)	0.411
Hypertension (%)	35 (58.3)	28 (54.9)	0.716
Malignance (%)	3 (5.0)	0 (0)	0.105
Heart disease (%)	12 (20.0)	12 (23.5)	0.653
Anemia (%)	1 (1.7)	4 (7.8)	0.118
Stroke (%)	15 (25.0)	9 (17.7)	0.348
Steroid use (%)	6 (10.0)	7 (13.7)	0.543
Smoking (%)	1 (1.7)	6 (11.8)	0.029^*∗*^
Preoperative hemoglobin level, g/L (mean ± standard deviation)	131.1 (12.6)	130.0 (13.5)	0.667
Diagnosis of RA (%)	3 (5.0)	2 (3.9)	0.785
Operation time, minutes (mean ± standard deviation)	120.7 (17.7)	128.2 (19.9)	0.038^*∗*^
Tourniquet time, minutes (mean ± standard deviation)	63.9 (11.1)	66.3 (11.3)	0.247
Intraoperative blood loss, ml (mean ± standard deviation)	238.2 (88.7)	244.3 (105.1)	0.739
Postoperative drainage, ml (mean ± standard deviation)	543.2 (339.8)	473.1 (335.5)	0.279
Blood transfusion (%)	26 (43.3)	36 (70.6)	0.004^*∗*^
Hypoxemia (%)	3 (5.0)	6 (11.8)	0.193

^*∗*^ *p* < 0.05 was considered statistically significant.

PPC, postoperative pulmonary complication; BMI, body mass index; RA, rheumatoid arthritis.

**Table 3:** Multivariate logistic regression analysis to identify independent risk factors for PPC.

	OR	95% CI	*p* value
BMI	0.879	0.776–0.995	0.042^*∗*^
Smoking	5.986	0.637–56.290	0.118
Operation time	1.023	0.999–1.047	0.057
Blood transfusion	2.616	1.134–6.038	0.024^*∗*^

^*∗*^ *p* < 0.05 was considered statistically significant.

OR, odds ratio; CI, confidence interval; BMI, body mass index.
